# Searching for a Longevity Food, We Bump into *Hericium erinaceus* Primordium Rich in Ergothioneine: The “Longevity Vitamin” Improves Locomotor Performances during Aging

**DOI:** 10.3390/nu14061177

**Published:** 2022-03-11

**Authors:** Elisa Roda, Daniela Ratto, Fabrizio De Luca, Anthea Desiderio, Martino Ramieri, Lorenzo Goppa, Elena Savino, Maria Grazia Bottone, Carlo Alessandro Locatelli, Paola Rossi

**Affiliations:** 1 Laboratory of Clinical & Experimental Toxicology, Pavia Poison Centre, National Toxicology Information Centre, Toxicology Unit, Istituti Clinici Scientifici Maugeri IRCCS, 27100 Pavia, Italy; elisa.roda@icsmaugeri.it (E.R.); carlo.locatelli@icsmaugeri.it (C.A.L.); 2 Department of Biology and Biotechnology “L. Spallanzani”, University of Pavia, 27100 Pavia, Italy; daniela.ratto01@universitadipavia.it (D.R.); fabrizio.deluca01@universitadipavia.it (F.D.L.); martino.ramieri01@universitadipavia.it (M.R.); mariagrazia.bottone@unipv.it (M.G.B.); 3 Department of Earth and Environmental Sciences, University of Pavia, 27100 Pavia, Italy; anthea.desiderio01@universitadipavia.it (A.D.); lorenzo.goppa01@universitadipavia.it (L.G.); elena.savino@unipv.it (E.S.)

**Keywords:** frailty, medicinal mushroom supplementation, *Hericium erinaceus*, primordium, oxidative stress, frail prevention, neuroprotection

## Abstract

Phenotypic frailty is characterized by a progressive decline in physical functioning. During ageing, morphological and functional alterations involve the brain, and chief theories involve oxidative stress, free radical accumulation, and reduced antioxidant defenses as the most implicated mechanisms. From boosting the immune system to fighting senescence, medicinal mushrooms have been found to have a number of health and longevity benefits. Among them, *Hericium erinaceus* (He) has been demonstrated to display a variety of physiological effects, including anti-aging properties. Thus, He represents an attractive natural source for developing novel medicines and functional foods, based on the identification of its active ingredients and metabolites. Particularly, *H. erinaceus* primordium (He2) extract contains a high amount of Ergothioneine (ERGO), the longevity vitamin. Herein, we revealed the preventive effect of ERGO-rich He2 extract in a preclinical model, focusing on locomotor decline during ageing monitored through spontaneous behavioral test. This effect was accompanied by a significant decrease in some oxidative stress markers (NOS2, COX2) paralleled by an increase in P53, showed in cerebellar cortex cells and fibres by immunohistochemistry. In summary, we demonstrated the neuro-protective and preventive effects of He2 extract during aging, probably due to its peculiarly high ERGO content.

## 1. Introduction

Aging is characterized by a progressive loss of physiological integrity, leading to gradual decline in physical and cognitive functions, due to different morphological and functional changes involving the brain, e.g., atrophy, oxidative stress, and reduced antioxidant mechanisms [1]. These typical hallmarks contribute to the aging process, together determining the aging phenotype and increasing vulnerability to death [2]. Currently, healthy aging emerged as a crucial issue due to the recent increase of the geriatric population worldwide [3,4]. Therefore, a great deal of effort to overcome age-related impairments is devoted to identifying potential risk factors together with novel therapeutic strategies, also non-pharmacological, and discovering natural substances that may prevent/revert the adverse effects of aging.

Recently, social isolation imposed by national and international health authorities to counteract the spread of the COVID-19 pandemic caused heavy effects, often accelerating physical and cognitive decline, depression, and sarcopenia in elderly frail people [5,6,7].

Reactive oxygen and nitrogen species (RONS), physiologically produced to a low extent during cellular metabolism, may interact with several biomolecules altering their function [8], particularly when they reach high levels. During aging, brain of frail mice tends to accumulate dysfunctional and aggregated proteins and mitochondria as a result of an oxidative imbalance: increased production of reactive oxygen species (ROS) and/or reduced antioxidant defenses. Typically, the cellular milieu of the brain exhibits signs of compromised bioenergetics, impaired neuroplasticity, inflammation, and accumulation of oxidatively modified molecules and organelles. These changes make the aging brain vulnerable to neurodegenerative disorders, e.g., Alzheimer’s and Parkinson’s diseases, and stroke [9].

Among oxidative stress mechanisms, aberrant production of nitric oxide (NO) in the brain characterizes pathological conditions such as neurodegenerative diseases, inflammation, and ischemia, and, conversely, oxidative stress has an imperative role in pathogenesis of cognitive impairments and development/progression of neural injury. Nitric oxide synthase (NOS) is the enzyme responsible for the conversion of L-arginine (Arg) to L-citrulline, which leads to NO generation [9,10].

Concerning other oxidative stress mediating factors, the cyclooxygenase 2 (COX2) enzyme overexpression appears to be a dual task event in the brain, acting as a marker and a crucial effector of neural damage after brain injuries and in physiological or pathological aging. Accordingly, the neuroprotective effect of COX2 inhibitors in the brain could be ascribable either to the reduction of prostanoid and free radical synthesis or to the arachidonic acid bearing to alternative metabolic pathways.

The arachidonic acid shunting postulate hypothesized that COX2 inhibitors’ neuroprotective effects could be mediated by enhanced formation of beneficial eicosanoids. In circumstances of COX2 inhibition, arachidonic acid accumulates or is converted to eicosanoids, known to have healthy effects in the brain. Arachidonic acid shunting and COX2 inhibition may be crucial to gain functional recovery after brain injuries, thus suggesting therapeutic implications other than suppression of prostaglandin synthesis and free radical formation [11].

*Hericium erinaceus* (*He*), also known as Lion’s mane and Monkey Head Mushroom [12,13] is an edible medicinal mushroom with many health properties [13], including antioxidant, anti-inflammatory, antisenescence, and nootropic effects in the Central Nervous System (CNS) [14,15,16,17,18,19]. Moreover, previous findings in mice demonstrated neuroprotective and anti-inflammatory action of *H. erinaceus.* In particular, experimental data demonstrated (i) the increase in neurotransmission between mossy fibers-CA3 synapse in hippocampus, (ii) the improvement in locomotor performances and recognition memory [20,21], and (iii) the bettering of recognition memory in aged frail mice, paralleled by hippocampal and cerebellar neurogenesis [22,23].

Ergothioneine (ERGO) is a thiol derivative of histidine, only obtained through dietary intake, and able to accumulate even at high concentration in some cells and tissues, owing to an organic cation transporter, namely OCNT1. Based on its demonstrated high affinity for ERGO, this receptor has been recently designated as the ERGO transporter (ETT) or even as SLC22A4, based on the name of the encoding gene [24,25,26]. Nonetheless, since most authors in the scientific community continue to use the term OCTN1, herein we still employ this nomenclature.

The de novo synthesis of ERGO has been described in various fungi, including edible genera of the Basidiomycota division, e.g., *Boletus* ssp., and *Actinobacteria*, but, notably, any ERGO synthesis has been detected in higher plants or animal species [25,27].

ERGO has been documented as a powerful antioxidant in vitro and in vivo. In particular, ERGO has been shown to be radioprotective, able to scavenge singlet oxygen, hydroxyl radical, hypochlorous acid, and peroxyls radicals, and also to inhibit peroxynitrite-dependent nitration of proteins and DNA [28]. Notably, ERGO has been demonstrated to attenuate oxidative stress and nitrosative damage induced by neurotoxic peptide also reducing apoptosis occurrence [29]. Further experimental evidences supported the role of ERGO in rescuing cells from stress-induced apoptosis, through the activation of an intracellular antioxidant pathway involving p38 MAPK genes cascade [30,31]. Recent in vivo findings corroborate the beneficial effects of ERGO able to reduce cisplatin-induced oxidative stress and histopathological changes, also mitigating apoptotic phenomena through the modulation of the pro-apoptotic protein p53 [32].

Dietary ERGO is rapidly and efficiently absorbed by OCTN1 from intestinal district and distributed to various body tissues, where it is highly retained [24,33,34,35], including the brain [35]. The absorption and retaining mechanism suggest that ERGO could have a useful function in the body. In particular, some studies demonstrated that, mediated by an increase in OCTN1, ERGO may be accumulated in injured tissue, characterized by high oxidative stress and inflammation levels [36,37,38], playing its potential cytoprotective action [25,36,38]. In particular, ERGO overloading has been demonstrated to protect neurons and preserved cognitive functions, following administration of neurotoxic compounds, e.g., b-amyloid, cisplatin, or D-galactose [27,30,31,39,40].

Previously, we described the presence of different nootropic metabolites, including ERGO, in the mycelium and sporophore of different strains of Italian *Hericium erinaceus*, demonstrating beneficial effects on cognitive and locomotor performances in a pre-clinical model of physiological ageing [22,23,41].

Recently, we bump into an *H. erinaceus* primordium, characterized by a high content in ERGO paralleled by the lack of Hericenones and erinacines [41]. Thus, we jumped at the opportunity to study the primordium sample in order to point out exclusive ERGO effect, identifying, for the first time, its specific preventive action on locomotor frailty.

To this aim, in the current investigation a multi-tiered approach was adopted for studying neuroprotective effects during ageing, starting by (i) monitoring spontaneous locomotor activity and measuring the frailty index and then (ii) evaluating specific aging-related biomarkers in CNS tissue using immunohistochemistry.

## 2. Materials and Methods

### 2.1. Animals and Treatments

**Figure 1:**
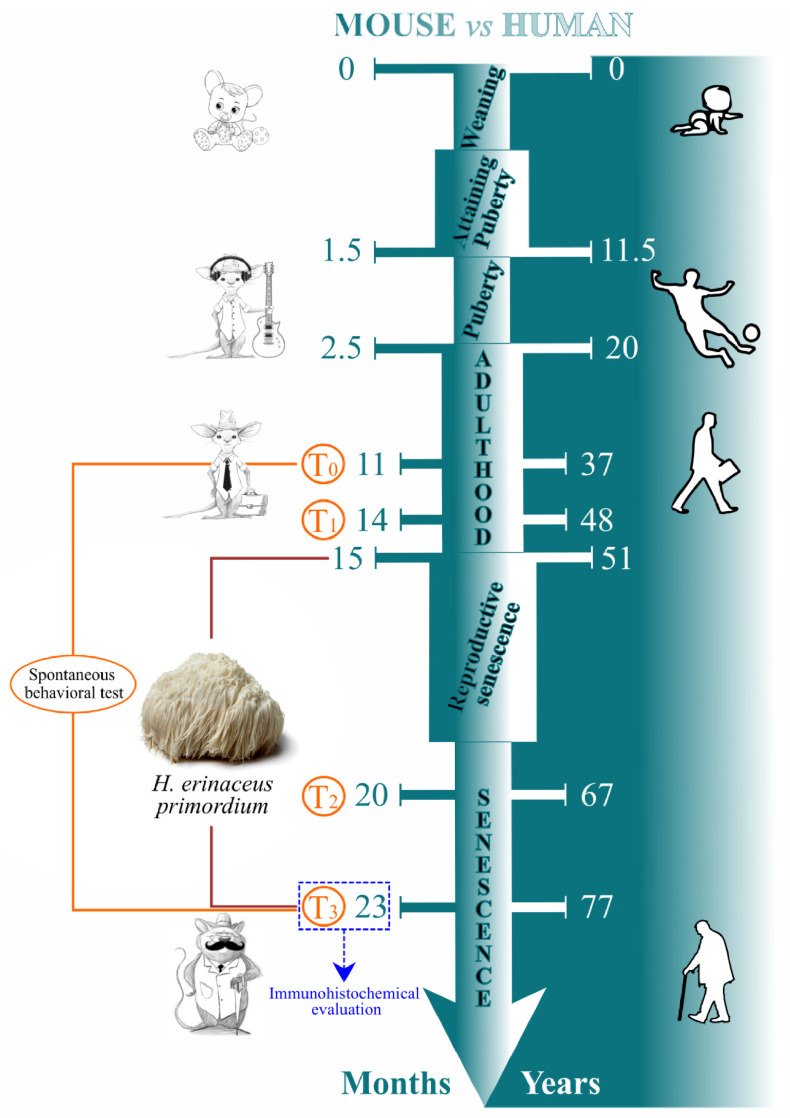
Schematic representation drawing experimental design (personalized from Dutta and Sengupta 2016, Ratto et al., 2019 [22,42]).

A total of fifteen pathogen-free C57BL-6J wild-type male mice (Charles River Italia, Calco, Italy) were acclimatized and maintained at the Animal Care Facility of the University of Pavia (maintenance parameters: 21 ± 2 °C, humidity at 50 ± 10%, 12 h light/dark cycle). Water and food were provided *ad libitum*. Experimental procedures were conducted in accordance with the guidelines laid out by the Ethics Committee of Pavia University (Ministry of Health, License number 774/2016-PR). Spontaneous behavioral tests were carried out at four different mice ages (corresponding to reported experimental times): 11 and 14 (T0 and T1, occurring during adulthood), 20 and 23 (T2 and T3, taking place during senescence) months old. At this latter timepoints, tissue sampling and immunohistochemical evaluation were also performed. Eight-month *H. erinaceus* oral supplementation: starting from 15 months of age, nine random mice were supplemented with a drink made with *H. erinaceus* (strain 2) primordium (namely He2) ethanol extracts solubilized in water at the final dose of 1 mg/day (namely P mice), whereas the remaining six mice did not receive any supplementation (namely C animals). The dose was selected to mirror human oral intake (1 g/day). Therefore, He2 primordium supplementation started during the adulthood phase and lasted until sacrifice at T3, during senescence phase. For experimental details see Figure 1.

Researchers conducting spontaneous behavioral tests, immunohistochemistry, and statistical analyses were blinded to the experimental condition (C and P mice).

### 2.2. Behavioral Test and Locomotor Frailty Index

Spontaneous behavioral test, namely open arena test, was performed for investigating locomotor performances of mice during time. The test was conducted as previously described [20,21]. During the test, mice were left free to explore an empty arena (63 × 42 cm), and their performances were recorded by a SMART video tracking system (2 Biological Instruments, Besozzo, Varese, Italy) and Sony CCD color video camera (PAL).

The table below (Table 1) report the selected locomotor parameters we focused on.

For each selected parameter, the corresponding locomotor frailty index (FI) was obtained using the following formula [22]:

FI = (Value−Mean Value at T0)/(SD at T0) × 0.25

Averaging the four selected locomotor parameters, we achieved the global locomotor frail index, FI, which defined the whole locomotor deterioration during senescent phase.

### 2.3. H. erinaceus Primordium

The He2 Italian strain was isolated from a wildtype sporophore collected in 2018 as previously described [43]. After aseptically cutting the sporophore core in small fragments (about 1 mm^3^), these portions were inoculated in Petri dishes containing 2% malt extract agar (MEA). The isolated strain was preserved in the MicUNIPV, the Fungal Research Culture Collection of University of Pavia. Through this isolation in pure culture, it was possible to cultivate the mycelium to obtain the sporophore, as previously reported [22]. At the early stages of growth, the primordium developed; it was collected, analyzed, and employed for the present research investigation.

#### 2.3.1. Extraction Procedures

The extraction procedure described by Bao et al. (2008) [44] was adopted and rearranged with the procedure previously reported in Corana et al. (2019) [41] and Ratto et al. (2019) [22] for other *H. erinaceus* metabolites, i.e., erinacines and hericenones. Specifically, 1 g of dried primordium of He2 was blended with ethanol 70% (10 mL), and then processed as detailly described in literature [22,45,46].

#### 2.3.2. HPLC-UV-ESI/MS Analytical Measurements

Chromatographic analyses were performed as previously reported [22]. In order to identify and measure the ERGO amount, He2 primordium extract was analyzed using HPLC-UV-ESI/MS, by comparison with ERGO standard solutions. L-(+)-Ergothioneine (497-30-3, TETRAHEDRON, Paris, France) was used as standard. The ERGO calibration curve was constructed by injecting five different concentrations of standard mixture solutions (10, 70, 150, 350 mg/L, analyzed in triplicate).

### 2.4. Cerebellum and Immunohistochemistry

For each group, cerebellar tissues were processed as detailly reported hereafter.

#### 2.4.1. Cerebellar Sampling

At T3 (23 months of age), mice were sacrificed and cerebella were immediately excised, fixed, and processed as previously described [47,48]. Eight micron-thick sections of cerebellar vermis were cut in the sagittal plane and collected on silane-coated slides.

#### 2.4.2. Immunohistochemistry by Light Microscopy

Immunohistochemical procedures were performed as previously described [23], using commercial antibodies on murine cerebellar specimens, to explore expression and distribution of specific molecules: (i) cyclo-oxygenase-2 (COX2), nitric oxide synthase 2 (NOS2), and (iii) p53. Cerebellar sections of C and P mice were incubated at RT overnight with PBS-diluted monoclonal and polyclonal primary antibodies (Table 2). In detail, COX2 and NOS2 were assessed being essentially involved in oxidative stress pathway [49,50,51,52]; p53 was assessed for its crucial role in cell death mechanism [53,54,55,56].

Proper biotinylated secondary antibodies (Table 2) and an avidin biotinylated horseradish peroxidase complex (Vector Laboratories, Burlingame, CA, USA) were employed to reveal the antigen/antibody interaction sites. The 3,3′-diaminobenzidine tetrahydrochloride peroxidase substrate (Sigma, St. Louis, MO, USA) was used as the chromogen. The nuclear counterstaining was achieved by employing Carazzi’s Hematoxylin. Then, the sections were dehydrated in ethanol, cleared in xylene, and finally mounted in Eukitt (Kindler, Freiburg, Germany). As negative untreated, some sections were incubated with PBS in the absence of the primary antibodies: no immunoreactivity was observed in this condition.

#### 2.4.3. Immunohistochemical Evaluations

Sections were observed in brightfield microscopy using an Olympus BX51 optical microscope (model BX51TF) and images were acquired with an Olympus CAMEDIA C4040ZOOM camera. For each investigated molecule, five slides (about 20 sections) per animal were analyzed. In both experimental groups, cerebellar samples with diverse immunolabeling degrees were considered. Figures show the most representative alterations for each immunohistochemical reaction. Immunohistochemical labeling extent was evaluated on acquired digitized section images under exposure time avoiding any pixel saturation effect. The labeling intensity was measured utilizing densitometric analysis (Image-J 1.48i; NIH, Bethesda, MA, USA) as previously reported in detail [23]. Specifically, the labeling was measured as the mean intensity value over the area, and the immunocytochemical intensity, namely OD, was evaluated in three randomized images/section per five slides/animal from each experimental group. Data were recorded on Microsoft Office Excel Software spreadsheets and the analysis was achieved using the ImageJ software.

#### 2.4.4. Statistics

Mean standard error of the mean (SEM) were reported. We performed Bartlett and ShapiroWilk Tests to establish and confirm parameters normality. Concerning behavioral tests, One-way Anova for repeated measures was used for investigating the aging effect (£ vs. 11 months (T0), # vs. 14 months; $ vs. 20 months), whereas two-way Anova was used to compare C and P groups. The differences were considered statistically significant for *p* < 0.05 (£, #, $, *), *p* < 0.01 (££, ##, $$, **), and *p* < 0.001 (£££, ###, $$$, ***). Concerning immunohistochemistry, the statistical analysis was carried out using an unpaired Student’s *t*-test. The differences were considered statistically significant for *p* < 0.05 (*), *p* < 0.01 (**), and *p* < 0.001 (***).

All statistical analyses were performed with GraphPad Prism 7.0 software (GraphPad Software Inc., La Jolla, CA, USA).

## 3. Results

### 3.1. ERGO Amount in He2 Primordium Extract

**Figure 2:**
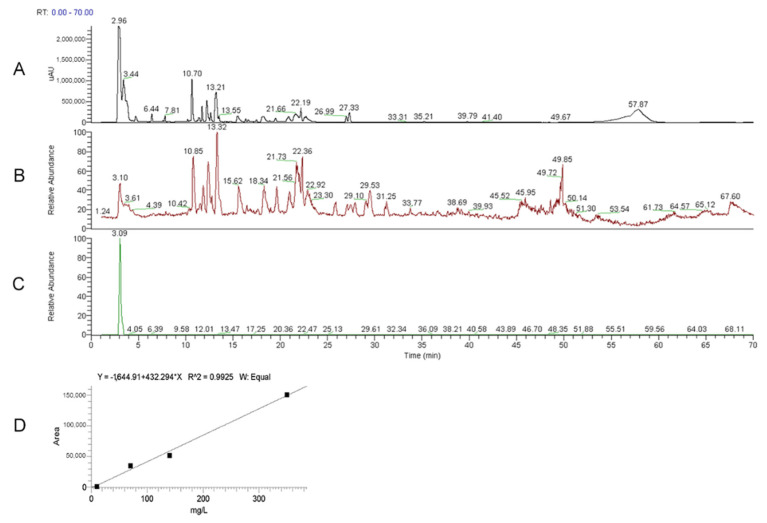
HPLC-UV-ESI/MS was used to quantify the ERGO amount in He2 extract. (**A**) UV (Ultra Violet) trace of He2 primordium. (**B**) ESI full MS (mass spectrum) trace of He2 primordium. (**C**) Standard molecule of L-(+)-ERGO: mass spectrum (MS)/MS Selected Reaction Monitoring *m*/*z* 230 > *m*/*z* 186 trace. (**D**) Calibration curves for ERGO.

Using HPLC-UV-ESI/MS, the presence of ERGO in the He2 primordium extract was assessed and then ERGO amount was measured by comparison with ERGO standard curve (Figure 2, panel D), as reported in Roda et al., 2021 [23]. Figure 2 shows the UV (panel A) and ESI full MS (mass spectrum) trace (panel B) of He2 primordium; panel C displays the standard molecule of L-(+)-ERGO in MS/MS Selected Reaction Monitoring *m*/*z* 230 > *m*/*z* 186 traces. Notably, ET was present in He2 sample with a measured content of 1.30 ± 0.57 mg/g.

### 3.2. Locomotor Decline during Physiological Aging and Its Partial Recovery by He2 Primordium Extract Oral Supplementation

**Figure 3:**
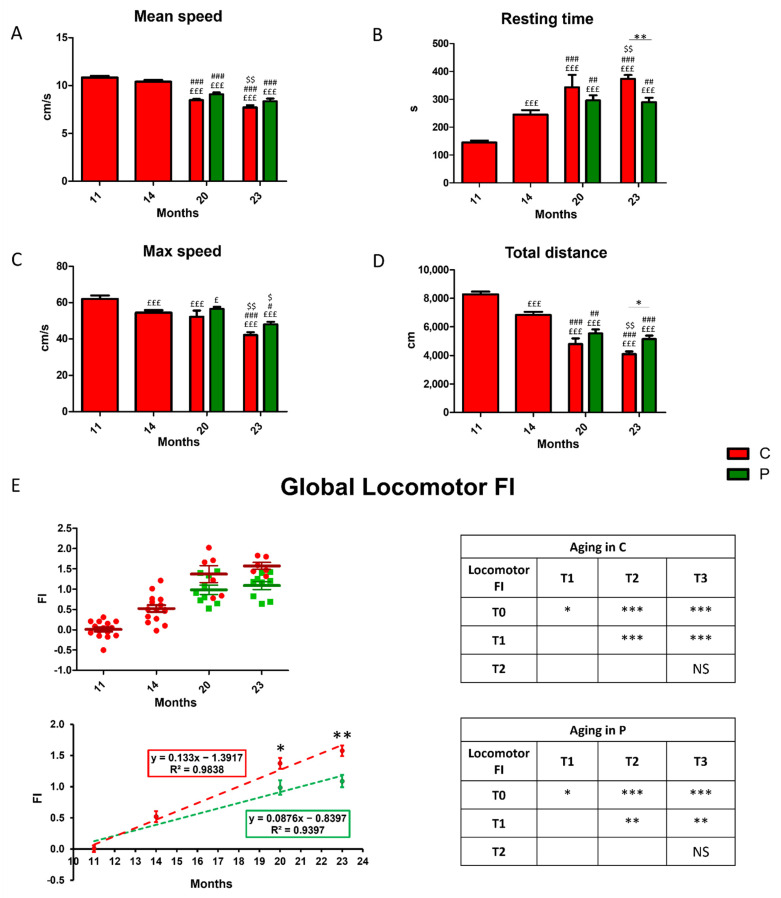
Decline of locomotor performances during physiological aging in control animals and recovery in He2 primordium extract-supplemented mice. (**A**): Mean speed (cm/s); (**B**): resting time (s); (**C**): max speed (cm/s); (**D**): total distance (cm). (**E**): Scatter plot of global locomotor FIs (up left), linear least-square regression of experimental points (down left) and separated tables showing statistical results regarding aging effect in wild-type controls (red, up right) and treated (green, down right) animals. For each panel, red bars or dots show data regarding control animals (C group), while green bars or dots show data belonging to treated mice (P group). Statistically significant values: *p* < 0.05 (^£, #, $,^ *); *p* < 0.01 (^##, $$,^ **); *p* < 0.001 (^£££, ###,^ ***).

Locomotor performances were evaluated by measuring selected parameters (Table 1, Section 2), recorded during the open arena test, at chosen timepoints, i.e., T0, T1, T2, and T3, corresponding to four different animal ages, i.e., 11, 14, 20, and 23 months. The first two experimental times occur during adulthood, whereas the last two take place during senescence. Firstly, we investigated the effects of physiological aging in non-supplemented wild-type mice, considered as controls, namely C animals (indicated by red bars and dots in Figure 3). Figure 3 illustrates the proceeding of locomotors parameters during aging. The mean speed (cm/s) significantly decreased starting from T2, then worsened over time (Figure 3A). A significant reduction of max speed (cm/s), and total distance (cm) paralleled by an increase of resting time (s), were measured already after T1 (Figure 3B–D).

Notably, the oral supplementation with He2 primordium extract, lasting for eight months (from 15 to 23), significantly improved locomotor performances in mice regarding the resting time and the total distance at T3, corresponding to 23 months of age (Figure 3C,D). A similar but non-significant trend was observed for the mean and maximum speeds.

Next, the locomotor FI was calculated for each parameter and, averaging the four locomotor FIs, we obtained a global Locomotor FI (Figure 3E). Notably, the global locomotor FI significantly augmented during aging both in controls (C, red dots) and treated (P, green dots) mice, even though this increase in P group was statistically lower compared to that measured in C animals. Indeed, the linear least-square regression for locomotor FIs analysis changed the slope from 0.133 for C group to 0.087 for P mice (Figure 3E).

### 3.3. Key Players of Oxidative Stress Pathway: COX2 and NOS2

Literature evidences emphasized the oxidative damage role during aging and its impairment due to an imbalance between reactive oxygen species (ROS) production and antioxidant scavengers system [57].

Currently, we immunohistochemically evaluated presence and specific localization of Cyclooxygenase 2 (COX2) and Nitric oxide synthase 2 (NOS2), as identifiable markers peculiarly implicated in oxidative stress pathway. Sagittal sections of the cerebellar vermis from both non-supplemented (C animals) and He2 primordium-treated mice (P group) at T3 (23-month-old mice) were used. The evaluations were focused on the posterior region (neocerebellar lobules VI–VIII), known to be mainly affected by age and correlated with motor function [23].

**Figure 4:**
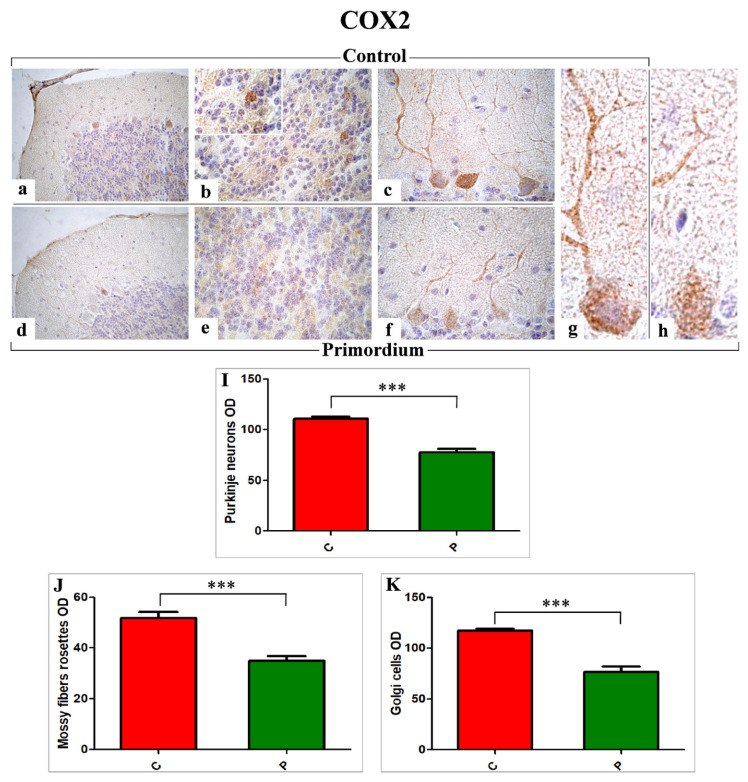
Immunohistochemical labelling for COX2 in C animals (**a**–**c**,**g**) and P (**d**–**f**,**h**) mice. Panels (**I**–**K**): Histograms showing the quantitative analysis of COX2 immunopositive PCs, mossy fiber rosettes, and GCs OD, respectively. *p* values calculated by unpaired Student’s *t*-test: *p* < 0.001 (***).

**Figure 5:**
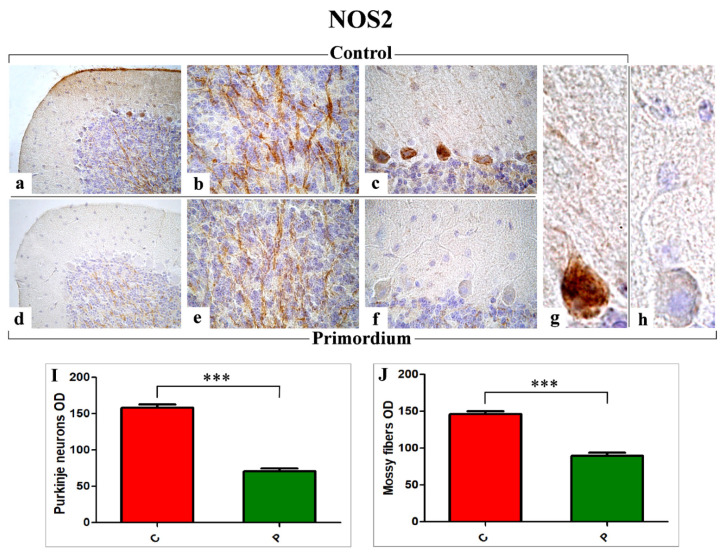
Immunohistochemical labelling for NOS2 in C (**a**–**c**,**g**) and P (**d**–**f**,**h**) mice. Panels I and J: Histograms showing the quantitative analysis of NOS2 immunoreaction evaluated as PCs and mossy fibers OD ((**I**) and (**J**), respectively)). *p* values, calculated by unpaired *t*-test, were considered significant for *p* < 0.001 (***).

A widespread expression and distribution of both markers, i.e., COX2 (Figure 4) and NOS2 (Figure 5), was detected in specific cerebellar cortex areas, i.e., Purkinje cells (PCs) and internal granular layer (IGL), both in C and P mice, but with different patterns. In particular, concerning COX2, somas of PCs exhibited a clear immunoreactivity. A strong immunoposiotivity was also detected in IGL, localized in the large mossy fiber rosettes and in several Golgi cells (GCs). These observed immunoreactivities appeared significantly more intense in C mice (Figure 4a–c,g) compared to P animals (Figure 4d–f,h). In line with the qualitative data, the successive quantitative analysis confirmed the significant decrease of COX2 immunoreactivity, evaluated in terms of cells/rosettes OD, in P mice compared to C animals (PCs: 77.66 ± 3.23 vs. 110.60 ± 2.51, mossy fibers rosettes: 34.94 ± 1.81 vs. 51.80 ± 2.35 and GCs: 76.73 ± 4.91 vs. 117.37 ± 1.89, respectively).

Regarding NOS2 (Figure 5), the immunohistochemical reactions revealed an evident immunoreactivity in the large soma of Purkinje neurons, more marked in C mice (Figure 5a–c,g) compared to P group (Figure 5d–f,h). Further, several heavily NOS2-immunopositive mossy fibers were observed in the IGL, showing the same intensity pattern already reported for PCs (Figure 5a–c,g vs. Figure 5d–f,h). Likewise, the analysis of NOS2-immunoreactivity OD, evaluated both at PCs soma and mossy fibers level, demonstrated a significant decrease in P animals compared to C mice (PCs: 70.73 ± 3.91 vs. 157.86 ± 4.85 and mossy fibers: 89.79 ± 3.71 vs. 146.01 ± 4.03, respectively).

### 3.4. p53 Immunohistochemical Assessment

**Figure 6:**
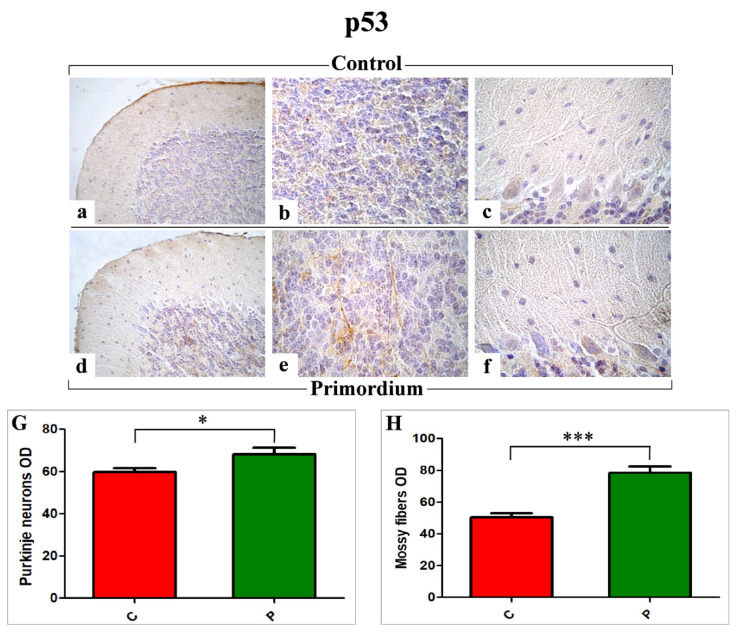
Immunohistochemical labelling for p53 in C (**a**–**c**) and P (**d**–**f**) mice. Panels G and H: Histograms showing the quantitative analysis of p53 immunopositive PCs and mossy fibers OD ((**G**) and (**H**), respectively). *p* values, calculated by unpaired *t*-test, were considered significant for *p* < 0.05 (*) and *p* < 0.001 (***).

Similar to the observed NOS2 immunoreactivity localization, the p53 immunopositivity (Figure 6) was detected both in PCs soma as well as in the IGL, where immunoreactive mossy fibers were observed. Nonetheless, notably, the p53 immunopositivity pattern was completely opposite compared to those evidenced for COX2 and NOS2, with the heaviest immunoreactivity observed in P mice (Figure 6d–f) compared to C group (Figure 6a–c). Accordingly, the following quantitative determination demonstrated an increased p53-immunoreactivity OD, evaluated both at PCs as well as at mossy fibers levels, in P mice compared to C animals (PCs: 68.28 ± 2.97 vs. 59.89 ± 1.68 and mossy fibers: 78.50 ± 3.93 vs. 50.57 ± 2.46, respectively) (Figure 6G,H).

## 4. Discussion

This is the first study describing the effect of a specific *H. erinaceus* primordium extract, namely He2 primordium, characterized by being enriched in ERGO, on locomotor activity and oxidative stress in murine brain. Mushroom primordium is formed by dense mycelial cords that develop with negative geotropism and consists of the transition stage between mycelium and sporophore. As previously described [41], HPLC-UV-ESI/MS analyses demonstrated that He2 primordium contains Hericenes A, B, C, and D, molecules structurally similar to Hericenones, but lacks in other metabolites, such as erinacine A, present in the mycelium, and Hericenone C and D, present in the sporophore.

In previous papers, we analyzed and quantified the content of erinacine A in mycelium and hericenones C, D, and ERGO in the sporophore of the Italian He1 [22,23], then correlated these contents with the capability to partially revert cognitive and locomotor frailty during ageing. In our last paper, we demonstrated the presence of ERGO and other nootropic metabolites in both the mycelium and sporophore of He1 [34].

In the present study, we determined that in He2 primordium, the amount of Hericenes A, B, C, and D was comparable to that measured in both the mycelium and sporophore. Diversely, the amount of ERGO was 0.58 mg/g in the mycelium, 0.34 mg/g in the sporophore [23], but notably, it was about 2–4-fold higher in the primordium, 1.30 ± 0.57 mg/g. Currently, evidences exist that only few foods can be enumerated based on their high ERGO concentration [58,59]. The relevant ERGO amount measured in our He2 primordium extract matches with recent data, demonstrating that fungi, particularly several medicinal mushrooms, are certainly the leading natural dietary source of ERGO [59].

We previously described in frail mice the effect of two-months oral supplementation of a mycelium and sporophore He1 blend [23] on locomotor activity during ageing. Frail mice were selected at 21.5 months in the senescence life span phase, and we demonstrated a partial recovery of locomotor performances as measured by the total frailty index that decreased by 10%. Herein, we studied the preventive effect on the frailty development during aging, starting with the primordium supplementation in the adulthood phase of the mice lifespan and maintaining this oral supplementation until the senescence phase. In this experimental condition we were able to prevent frailty at 20 and 23 months in the senescence phase showing a frailty index, on the mean, less than 1, value chosen for convention as the threshold to define frail animals.

He2 primordium supplementation decrease the locomotor frailty index from 1.5 to 1, with a decrease of 43.45%. Given this data, and comparing with the effects previously described [22], we hypothesize that He2 primordium-induced preventive efficacy on the locomotor activity during ageing, could be attributed to its peculiar high concentration of ERGO.

A bulk of literature documented the role of ERGO as a powerful player in the oxidant/antioxidant balance both in vitro and in vivo, executing its valuable antioxidant cytoprotective action [25,28,29,36,37,38].

Oxidative stress impairs healthful aging, and accordingly, protection against oxidative stress is the key mechanism to be exploited to achieve the phenotype observed in animal models of longevity [56]. A body of literature undeniably confirmed that oxidative damage is crucially involved in age-associated cognitive and locomotor decline. In particular, aged neurons gather impaired/aggregated proteins and damaged mitochondria due to oxidative stress. Parallelly, the enhanced production of ROS together with the decrease in antioxidant scavengers represent the key participants in this unbalanced process, leading to senescence [54]. Thus, the normal antioxidant defense mechanisms break down, rendering the aged brain vulnerable to the consequences of oxidative stress [23,54,56,57,60,61]. Several studies confirmed the lessening of oxidative damage paralleled by the enhanced resistance to oxidative stress in longer-lived animals, gained via nutritional limitation or genetic manipulations allowing increased natural lifetime [53,54,56]. Remarkably, concerning CNS, recent data supported the hypothesis that stress-related events are characterized by modifications of oxidative/nitrosative pathways in the brain in response to the activation of inflammatory mediators. A key role for NO and an excess of pro-oxidants in different brain areas have been confirmed as responsible for both neuronal functional impairment and structural injury. NOS2 has been implicated in CNS cellular toxicity, and it has been verified that stress phenomena induce NOS2 expression in rodent brain and, conversely, its inhibition protects against ischemia-injury induced neuronal cell death [17]. NOS2 activation and overexpression is followed by large amounts of oxygen and nitrogen reactive species leading to the oxidation of cell constituents. Moreover, brain volume reduction together with BBB impairment have been described in the senescent brain, in major depressive syndromes, and in neurodegenerative disorders, as a possible consequence of oxidative stress imbalance [19,23,62]. On the other hand, COX pathway, depending on NFκB activation, has also been implicated in stress-induced brain damage. Both enzymatic sources of oxidative mediators in the brain is governed by the activation of the NMDA glutamate receptors, even though a role for TNF-α release has been also confirmed. These data clearly indicated that excitatory amino acids, and successive NFκB activation, determined stress-induced NOS2 and COX2 expression/activity in the brain [62]. Notably, many studies associated oxidative stress with inflammation, showing inflammation-mediated oxidative damage and emphasized the role of oxidative stress a critical mechanism linking inflammation, and cell injury/death; nonetheless, the complex relationship between oxidative stress and inflammation still remains a highly argued issue [23].

In line with literature, our current findings proved the protective role of the eight-months oral supplementation with He2 primordium, triggering a partial oxidative stress recovery in aged mice. In particular, we demonstrated a significant decrease of COX2 and NOS2 in supplemented animals, paralleled by an enhancement of p53 levels. These changes in molecules pivotally involved in aging could possibly increase lifetime or even ameliorate life quality together with an improvement in locomotor performances.

Our data are in accordance with previous literature findings revealing that natural extract-enriched diet diminished age-associated COX2 and NOS2 expressions augments [63]. Concerning the oxidative stress pathway, our results proved a marked reduction of both NOS2 and COX2 immunopositivity in P mice only, paralleled by a simultaneous increase of p53 immunoreactivity. In particular, compared to previous data [23], different immunoreactivity pattern and immunopositive cellular types and fibers were observed. Moreover, it has to be highlighted that our present findings demonstrated that the eight-months oral supplementation with He2 primordium extract enriched in ergothioneine was able to trigger more striking effects in aged mice, in terms of heavier extent and wider localization, compared to those observed in our previous study testing the beneficial effects of mycelium and sporophore extracts of He1. Specifically, concerning COX2, the marked immunoreactivity was detected not only in PCs, as previously determined in supplemented mice, but even in Golgi cells as well as in the mossy fiber rosettes. As regarding NOS2, our previous results demonstrated the lessening of immunopositivity mainly localized in PCs soma and mossy fibers rosettes in He1 treated mice. Presently, we proved, in P mice only, a stronger reduction of NOS2 immunopositivity, displaying a partially different localization, being chiefly detected in PCs as well as in mossy fibers, with the mossy fiber rosettes completely unreactive.

These results related to oxidative stress pathways were further corroborated by data on p53, known to play a role as crucial coordinator of oxidative stress and aging [53,54,55,56].

In response to a multitude of stressors, including oxidative stress, p53 is activated as a transcription regulator, leading to a quick accumulation of itself in stressed cells. Moreover, p53 is able to influence a variety of molecular pathways through posttranslational modifications, including protein phosphorylation, acetylation, methylation, and ubiquitination. Literature evidence corroborated the environment-depending role of p53, which exhibits antioxidant activities, to ensure cell survival, in response to oxidative stress low levels, while, contrarily, revealing prooxidative activities in response to high levels of oxidative stresses, when it promotes a further increase of stress levels, leading to cell death. Many studies suggested that oxidative stress may elicit a specific p53 transcriptional response to control cellular senescence and aging [53,55].

However, it has to be taken into careful consideration that oxidative stress conditions do not always provoke cell aging, since evidence exists that when the oxidative unbalance is mild, p53 is able to induce the expression of antioxidant genes to prevent cell death. By contrast, enhanced p53 levels could accelerate the generation of ROS and induce cell death under severe cellular stress. Nonetheless, notably, ex vivo studies reported that enhanced p53 levels in murine stem cells, isolated from transgenic mice with higher p53 activity, was associated with a slower rate of cell proliferation but a relatively younger status at a molecular level. Moreover, in vivo investigations demonstrated that p53-overexpressing transgenic mice did not display any signs of accelerated aging. Other in vivo evidence indicated that (i) transgenic mice with a hypomorphic mutation in Mdm2 exhibited increased p53 activity but normal life span and (ii) mice with an additional copy of p53 showed an enhanced expression of antioxidant activity and decreased levels of endogenous oxidative stresses, correlating with an increased life span [53,54,56].

Taken together, these controversial data support the notion that the precise role of p53 in cell aging is complex and strictly linked to the environmental situation. In this context, the p53 activation in response to low oxidative stress could protect cells against oxidative damage, thus supporting the role of p53 in the maintenance of tissue homeostasis.

In line with these results, our findings, revealing an increase of p53 in P mice, in which a decrease of COX2 and NOS has contextually been demonstrated, led us to hypothesize that the p53 enhancement could prevent the accumulation of oxidative stress molecules at high levels, inhibiting senescence progression, thus achieving a delayed aging in these mice.

## 5. Conclusions

**Figure 7:**
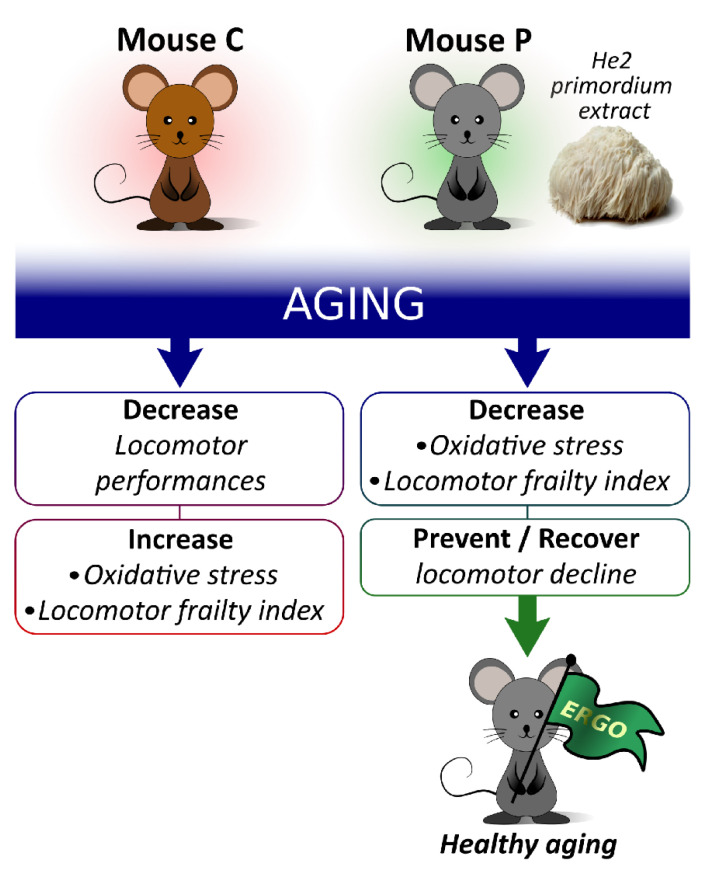
Pictorial illustration summarizing main results and take-home message.

He2 primordium extract acts as a valuable candidate to prevent and partially recover locomotor decline, contextually lowering oxidative stress during aging. We assume that this neuro-protective and preventive effect could be ascribable to its peculiarly high ERGO content. In particular, ERGO could exert its potential to ameliorate/mitigate/revert aged-dependent impairments, thereby increasing life quality and expectancy when taken in adequate amounts (Figure 7). Our findings substantiate the use of He2 primordium extract as a novel therapeutic strategy to be used in geriatrics settings, including nursing homes, to prevent or treat aged-related diseases, improving the elderly quality of life. Further studies are needed to exploit He2 primordium potential effects on the cognitive decline as well as the implicated cellular mechanisms. Our final goal will be to translate experimental findings to clinical settings, being able to propose an effective natural adjuvant therapy to be used in geriatric patients’ management.

## Figures and Tables

**Table 1:** Open arena test and chosen parameters.

Spontaneous Behavioral Test	Selected Locomotor Parameters
Open arena	Resting Time (s)
	Total Distance (cm)
	Max Speed (cm/s) Mean Speed (cm/s)

**Table 2:** Primary and secondary antibodies used for immunocytochemistry.

	Antigen	Immunogen	Manufacturer, Species, Mono-Polyclonal, Cat./Lot. No., RRID	Dilution
Primary antibodies	Anti-Nitric Oxide Synthases-2 (M19)	Purified antibody raised against a peptide mapping at the C-terminus of NOS2 of mouse origin	Santa Cruz Biotechnology (Santa Cruz, CA, USA), Rabbit polyclonal IgG, Cat# sc-650, RRID: AB_631831	1:100
	Anti- Cyclooxygenase-2 (M-19)	Purified antibody raised against a peptide mapping at the C-terminus of COX2 of mouse origin	Santa Cruz Biotechnology (Santa Cruz, CA, USA), Goat polyclonal IgG, Cat# sc-1747, RRID: AB_2084976	1:100
	Anti-p53 (Ab-5)	Purified antibody raised against the ~53 kDa wild type p53 protein of mouse origin	Sigma-Aldrich (St. Louis, MO, USA), Mouse monoclonal IgG2a, Cat# OP33-100UG, RRID: AB_564977	1:100
Secondary Antibodies	Biotinylated horse anti-mouse IgG	Gamma immunoglobulin	Vector Laboratories (Burlingame, CA, USA), Horse, Cat# PK-6102, RRID: AB_2336821	1:200
	Biotinylated goat anti-rabbit IgG	Gamma immunoglobulin	Vector Laboratories (Burlingame, CA, USA), Goat, lot# PK-6101, RRID: AB_2336820	1:200
	Biotinylated rabbit anti-goat IgG	Gamma immunoglobulin	Vector Laboratories (Burlingame, CA, USA), Rabbit, Cat# PK-6105, RRID: AB_2336824	1:200

## Data Availability

Not applicable.
